# Rucksack palsy after military boot camp

**DOI:** 10.1590/0004-282X-ANP-2021-0427

**Published:** 2022-08-08

**Authors:** Pedro Henrique Almeida Fraiman, Igor Matheus Diniz Papa, Bruno de Medeiros Fernandes, Fernanda Thaysa Avelino dos Santos, Clecio Godeiro-Junior

**Affiliations:** 1 Universidade Federal de São Paulo, Escola Paulista de Medicina, Departamento de Neurologia, São Paulo SP, Brazil.; 2 Hospital de Guarnição de Natal, Exército Brasileiro, Natal RN, Brazil.; 3 Universidade Federal do Rio Grande do Norte, Hospital Universitário Onofre Lopes, Divisão de Neurologia, Natal RN, Brazil.

**Figure 1.**
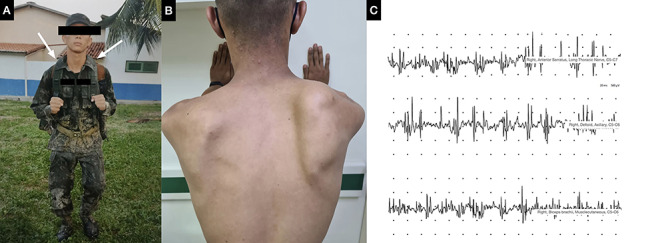
Rucksack palsy. (A) Military rucksack carries a load of approximately 15 kilograms; superior armpit is a point of compression (white arrows). It is the result of traction of brachial nerves on scalene muscles caused by the shoulder straps of a heavy rucksack. (B) Hypotrophy of right shoulder girdle muscles, more prominent deltoid, supraspinatus, biceps brachii muscles, and winged scapula. (C) Electroneuromyography signs of progressive subacute neurogenic motor unit potentials of right deltoid, biceps brachii, anterior serratus suggesting pathology of the brachial plexus.

A 18-year-old soldier reported weakness on the abduction of right arm and mild right shoulder pain five days after wearing a rucksack during a 3-day boot camp training (Figure 1-A). After six weeks, he presented hypotrophy of shoulder girdle muscles and winged scapula (Figure 1-B). An electroneuromyography performed at this moment revealed signs of progressive subacute neurogenic motor unit potentials of right deltoid, biceps brachii and anterior serratus (Figure 1-C). Right brachial plexus, shoulder MRI and a viral serum panel were unremarkable. 

Rucksack palsy is most described in the military population^1^. It is associated with damage to the brachial plexus as a result of wearing a heavy rucksack^2^. 
